# Differential Epigenetic Signature of Corticospinal Motor Neurons in ALS

**DOI:** 10.3390/brainsci11060754

**Published:** 2021-06-07

**Authors:** Tunch Ozyurt, Mukesh Gautam

**Affiliations:** Davee Department of Neurology and Clinical Neurological Sciences, Northwestern University Feinberg School of Medicine, Chicago, IL 60611, USA; tunch.ozyurt@northwestern.edu

**Keywords:** CSMN, ALS, 5-methylytosine, 5-hydroxymethycytosine, epigenetics, mSOD1, TDP-43

## Abstract

Corticospinal motor neurons (CSMN) are an indispensable neuron population for the motor neuron circuitry. They are excitatory projection neurons, which collect information from different regions of the brain and transmit it to spinal cord targets, initiating and controlling motor function. CSMN degeneration is pronounced cellular event in motor neurons diseases, such as amyotrophic lateral sclerosis (ALS). Genetic mutations contribute to only about ten percent of ALS. Thus understanding the involvement of other factors, such as epigenetic controls, is immensely valuable. Here, we investigated epigenomic signature of CSMN that become diseased due to misfolded SOD1 toxicity and TDP-43 pathology, by performing quantitative analysis of 5-methylcytosine (5mC) and 5-hydroxymethycytosine (5hmC) expression profiles during end-stage of the disease in hSOD1^G93A^, and prpTDP-43^A315T^ mice. Our analysis revealed that expression of 5mC was specifically reduced in CSMN of both hSOD1^G93A^ and prpTDP-43^A315T^ mice. However, 5hmC expression was increased in the CSMN that becomes diseased due to misfolded SOD1 and decreased in CSMN that degenerates due to TDP-43 pathology. These results suggest the presence of a distinct difference between different underlying causes. These differential epigenetic events might modulate the expression profiles of select genes, and ultimately contribute to the different paths that lead to CSMN vulnerability in ALS.

## 1. Introduction

Corticospinal motor neurons (CSMN) are a unique population of motor neurons that are important for motor function. These neurons collect and integrate inputs from other neurons in the brain, and relay that information to their spinal cord targets, thus maintaining a functional motor neuron circuitry [1]. Degeneration of CSMN results in motor neuron diseases such as hereditary spastic paraplegia (HSP), primary lateral sclerosis (PLS), and amyotrophic lateral sclerosis (ALS) [2]. ALS is a complex and heterogeneous disease that is manifested by progressive degeneration of CSMN, and spinal motor neurons [3]. ALS can be hereditary as well as sporadic, with a major contribution from sporadic factors. CSMN loss is detected in both familial and sporadic forms of ALS [4,5]. Early detection of CSMN loss is beginning to emerge as an early diagnostic marker of ALS [6], and improving CSMN health proved to be beneficial for the health and integrity of motor neuron circuitry [7]. 

There are about 147 genes mutated in ALS patients that are either causative or associated with ALS [8]. Many of these genes converge on specific cellular defects [8], two of which are prominent in ALS; the mutant SOD1 mediated toxicity and TDP-43 pathology mediated cell death. Among the mouse models generated to understand the disease mechanisms related to SOD1 toxicity and TDP-43 pathology, we find hSOD1^G93A^ and prpTDP-43^A315T^ to be well characterized, and most importantly to faithfully recapitulate ALS pathology at a cellular level [9,10]. Involvement of mSOD1 in ALS pathology is widely studied, and hSOD1^G93A^ mouse shows progressive degeneration of CSMN [11]. Mis-localization and accumulation of TDP-43 protein in the cytoplasm is the most common ALS pathology that is detected in both, sporadic and familial forms of ALS [12]. prpTDP-43^A315T^ mouse model display progressive degeneration of CSMN due to defect in cellular organelles such as mitochondria, ER, and nuclear membrane. Similar cellular defects are also observed in the upper motor neurons of ALS patients with TDP-43 pathology [13]. Interestingly, SOD1 toxicity and TDP-43 pathology have unique and non-overlapping aspects of neurodegeneration. For example, patients with SOD1 mutation did not have TDP-43 pathology in their CNS, even though TDP-43 accumulations are widely observed in a broad spectrum of ALS patients [14]. Similarly, the mouse models also show similar findings, such that ALS mouse models expressing different mutations in SOD1 gene (hSOD1^G93A^, hSOD1^G37R^ and hSOD1^G85R^) did not show TDP-43 accumulation [15]. Therefore, investigating epigenetic modification in these two distinct disease models will be immensely valuable in understanding selective vulnerability of CSMN. 

Epigenetics is the modulation of gene expression without any changes in the gene sequence. Expression of genes can be modulated via two epigenetic mechanisms: DNA methylation, and DNA hydroxymethylation [16]. Addition of methyl group at 5 carbon position on cytosine by DNA methyltransferase (Dnmt) enzyme converts cytosine into 5-methylcytosine (5mC) by transfer of a methyl group from *S*-adenosyl-methionine [17]. 5-hydroxymethylcytosine (5hmC) is generated through the oxidation of 5mC by the ten-eleven translocation (TET) family of methylcytosine dioxygenases [18,19]. These epigenetic modifications alter gene expression via differential methylation or hydroxymethylation of promoter regions of a defined population of genes. DNA methylation/hydroxymethylation changes protein–DNA interactions that lead to alterations in chromatin structure and rate of transcription [20]. A growing body of evidence suggests key role of epigenetic mechanisms in ALS pathology [21]. Global epigenome-wide studies of cortex, and spinal cord of sALS patients have shown differential methylation and hydroxymethylation of a select set of genes [22,23]. Recently, TDP-43 pathology was shown to be associated with DNA methylation [24]. Methylation of *C9orf72* promoter is reduced while hydroxymethylation is increased during differentiation of ALS patient-derived iPS cells into motor neurons [25]. Additionally, promotor region of *C9orf72* have shown increased methylation and decreased transcription in ALS/FTD patients with the pathogenic repeat expansion [26,27,28]. 

The role of epigenetic modifications, and their contribution to ALS pathogenesis is beginning to emerge. However, CSMN specific epigenetic modifications have not been investigated thus far. This study was designed to investigate whether DNA methylation, and hydroxymethylation levels in CSMN are differentially modulated in the context of ALS. Using hSOD1^G93A^ and prpTDP-43^A315T^ mouse models of ALS, which represent disease mechanisms that occur due to misfolded SOD1 toxicity and TDP-43 pathology, respectively, here, we reveal that CSMN based on the underlying cause of the disease display a distinct pattern of methylation and hydroxymethylation. Epigenetic modifications are an important event in the regulation of gene expression. We find that CSMN that are diseased due to mSOD1 toxicity, and TDP-43 pathology have distinct pattern of DNA methylation/hydroxymethylation status. These results begin to reveal the epigenetic regulation of CSMN vulnerability in ALS.

## 2. Materials and Methods

### 2.1. Mice

All animal experiments were performed in compliance with the standards set by National Institutes of Health (NIH) and were approved by the Northwestern University Animal Care and Use committee (NUACUC). The following mouse strains were used in this study: prp-TDP-43^A315T^ (procured from Jackson Laboratory, stock no. 010700), hSOD1^G93A^ (procured from Jackson Laboratory, stock no. 002726), and UCHL1-eGFP (generated by the Ozdinler Lab at Northwestern Targeted Mutagenesis Core Facility, now also available at Jackson Laboratory, stock no. 022476). UCHL1-eGFP is a reporter mice line in which CSMN are specifically labeled with eGFP allowing their precise visualization and investigation [29]. Hemizygous UCHL1-eGFP females were bred to hemizygous prp-TDP-43^A315T^ males to generate prp-TDP-43^A315T^-UeGFP mice. Similarly, hemizygous UCHL1-eGFP females were bred to hSOD1^G93A^ males to generate hSOD1^G93A^-UeGFP mice. The analyses were performed at the end stage of hSOD1^G93A^-UeGFP (P120) and prp-TDP-43^A315T^-UeGFP (P150) mice, respectively. All the mice used in this study were on C57/BL6 background. 

### 2.2. Tissue Collection and Processing

Mice were deeply anesthetized with intraperitoneal injection of ketamine (90 mg/kg, and xylazine (10 mg/kg; Fort Dodge Animal Health, Fort Dodge, IA, USA) prior to transcardial perfusion with phosphate-buffered saline (PBS) and 4% paraformaldehyde (PFA) in PBS. Intact brain was dissected, post-fixed in 4% PFA overnight and stored for later use in PBS with 0.01% sodium azide at 4 °C. The brain was sectioned at 50 μm using Leica vibratome (Leica VT1000S, Leica Inc., Nussloch, Germany).

### 2.3. Immunocytochemistry

Before commencing antibody incubation, floating sections were treated with 4N hydrochloric acid for 30 min at room temperature (RT) to denature the DNA and to facilitate the antibody penetration. Sections were washed three times with PBS for 10 min followed by incubation in blocking solution (PBS, 0.05% bovine serum albumin, 2% fetal bovine serum, 1% Triton X-100 and 0.1% saponin) for 30 min at RT prior to addition of primary antibodies: chicken anti-GFP (1:1000, Abcam, Cambridge, MA, USA), mouse anti-5-Methylcytosine (1:500, EpiGentek, Farmingdale, NY, USA), and mouse anti-5-Hydroxymethylcytosine (1:500, EpiGentek, Farmingdale, NY, USA) at 4 °C overnight. Excess antibodies were removed by PBS washes (three washes, 10 min each wash), sections were incubated in appropriate secondary antibodies in blocking solution: anti-chicken Alexa Fluor 488 (1:1000, Thermo Fisher Scientific, Rockford, IL, USA), anti-mouse Alexa Fluor 568 (1:1000, Thermo Fisher Scientific, Rockford, IL, USA) for 2 hrs at RT. Sections were counterstained with DAPI (1:5000). All sections were mounted on slides and coverslipped with Fluoromount G (Electron Microscopy Sciences, Hatfield, PA, USA). All immunostaining were performed at the same time with the same antibody cocktail to reduce experimental variation. 

### 2.4. Imaging

Nikon Eclipse TE2000-E (Nikon Inc., Melville, NY, USA) and Zeiss 880 confocal microscope (Carl Zeiss microscopy, Jena, Germany) were used to acquire low-and high-magnification images, respectively. Z-stacks were processed using ImageJ (National Institutes of Health, Bethesda, MD, USA, http://imagej.nih.gov/ij accessed on 16 April 2021) to generate maximum intensity projections.

### 2.5. Quantification and Data Analysis

CSMN from WT-UeGFP, hSOD1^G93A^-UeGFP, and prp-TDP-43^A315T^-UeGFP mice (*n* = 3) expressing 5mC, and 5hmC were counted using maximum projection images acquired from 50-μm thick sections. An equivalent area of the motor cortex (1.55 mm × 1.55 mm area corresponding to the 10× objective field size) was defined in three serial sections (~600 μm apart) per mouse. Neurons with pyramidal morphology and a visible apical dendrite were counted in each area, with experimenter blind to age and genotype of mice.

For intensity measurement, a maximum intensity projection image was opened using ImageJ (NIH), and CSMN were traced using free hand tool. Intensity of signals was measured in the defines area, and integrated density was recorded. A similar area was drawn in the background where there were no signals present. The integrated density of background was deducted from the integrated density of signal to measure the intensity of expression of 5mC, and 5hmC.

### 2.6. Statistical Analyses

GraphPad Prism software (GraphPad Software Inc., La Jolla, CA, USA) was used for statistical analyses. At least *n* = 3 mice and *n* = 3 brain sections for each mouse were used for each genotype and group. D’Agostino and Pearson normality tests were performed on all data sets. Student’s *t* test was used to determine statistical differences between experimental groups depending on the genotype, experimental conditions, and the disease group. Data are shown as mean ± SEM of at least three replicates and are representative of three independent experiments unless otherwise stated and statistically significant differences were taken at *p* < 0.05, and *p* values are reported in the text. 

## 3. Results

### 3.1. CSMN Diseased Due to mSOD1 Toxicity and TDP-43 Pathology Displayed Reduced Methylation

**Figure 1:**
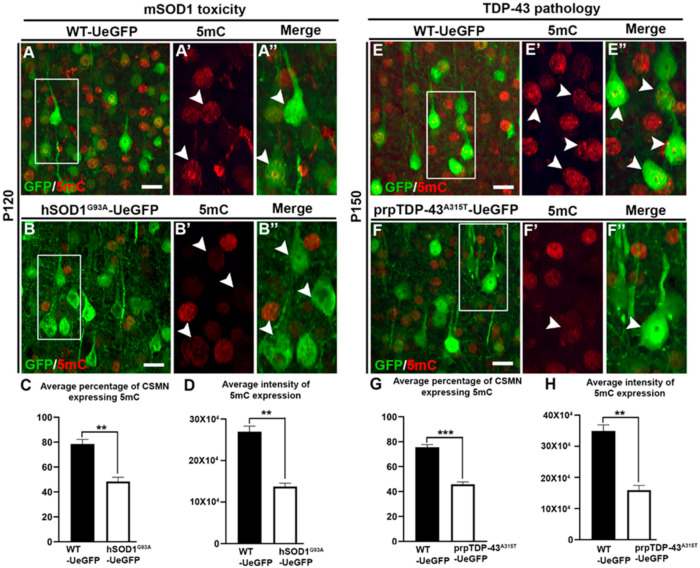
CSMN that are vulnerable in ALS due to different underlying causes display reduced levels of 5-methylcytosine (5mC). (**A**) Representative image of layer V of motor cortex from WT-UeGFP mouse at P120 showing GFP labelled CSMN and 5mC expression. Box enlarged (**A′**,**A″**, arrow heads). (**B**) Representative image of layer V of motor cortex from hSOD1^G93A^-UeGFP mouse at P120 showing GFP labelled CSMN and 5mC expression. Box enlarged (**B′**,**B″**, arrow heads). (**C**) Quantitative analysis of average percentage of CSMN expressing 5mC in hSOD1^G93A^-UeGFP mouse at P120. (**D**) Quantitative analysis of average intensity of 5mC expression in CSMN of hSOD1^G93A^-UeGFP mouse at P120. (**E**) Representative image of layer V of motor cortex from WT-UeGFP mouse at P150 showing GFP labelled CSMN and 5mC expression. Box enlarged (**E′**,**E″**, arrow heads). (**F**) Representative image of layer V of motor cortex from prp-TDP-43^A315T^-UeGFP mouse at P150 showing GFP labelled CSMN and 5mC expression. Box enlarged (**F′**,**F″**, arrow heads). (**G**) Quantitative analysis of average percentage of CSMN expressing 5mC in prp-TDP-43^A315T^-UeGFP mouse at P150. (**H**) Quantitative analysis of average intensity of 5mC expression in CSMN of prp-TDP-43^A315T^-UeGFP mouse at P150. ** *p* < 0.001, *** *p* < 0.0005; Scale bar: 20 µm.

Methylation status of CSMN was analyzed to investigate whether vulnerable CSMN have increased or decreased methylation levels. We used expression of 5-methylcytosine to determine the methylation levels in CSMN of hSOD1^G93A^ and prp-TDP-43^A315T^ mouse models of ALS, because progressive CSMN degeneration is well characterized in these two mouse models that represent two different underlying causes of the disease [11,13]. Analysis of CSMN expressing 5mC showed that most CSMN failed to express 5mc in hSOD1^G93A^ mouse (WT-UeGFP: 78.5 ± 3.7%; hSOD1^G93A^-UeGFP: 48.4 ± 3.41%, *n* = 3, *p* < 0.003, Figure 1A–C), and even the CSMN which continued to express 5mC had less fluorescence intensity (WT-UeGFP: 269,717 ± 13,116; hSOD1^G93A^-UeGFP: 137,023 ± 8441, *n* = 3, *p* < 0.001, Figure 1D). CSMN which become diseased due to TDP-43 pathology also showed less methylation (WT-UeGFP: 75.6 ± 2%; prp-TDP-43^A315T^-UeGFP: 45.8 ± 1.9%, *n* = 3, *p* < 0.0005, Figure 1E–G), and decreased levels of fluorescent intensity for 5mC expression (WT-UeGFP: 349,130 ± 19,675, prp-TDP-43^A315T^-UeGFP: 159,238 ± 15,042, *n* = 3, *p* < 0.001, Figure 1H).

### 3.2. CSMN Show Increased Hydroxymethylation Due to mSOD1 Toxicity, but Decreased Hydroxymethylation Due to TDP-43 Pathology

**Figure 2:**
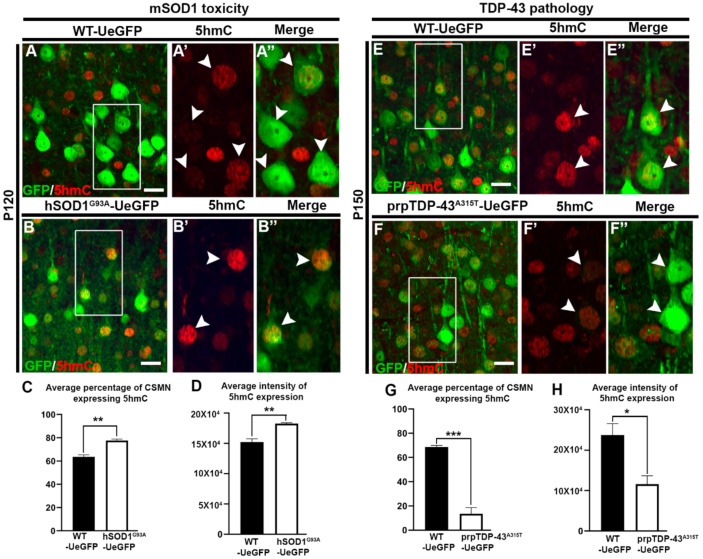
CSMN that are diseased owing to mSOD1 toxicity, and TDP-43 pathology display differential expression of 5-hydroxymethylcytosine (5hmC). (**A**) Representative image of layer V of motor cortex from WT-UeGFP mouse at P120 showing GFP-labelled CSMN and 5hmC expression. Box enlarged (**A′**,**A″**, arrow heads). (**B**) Representative image of layer V of motor cortex from hSOD1^G93A^-UeGFP mouse at P120 showing GFP-labelled CSMN and 5hmC expression. Box enlarged (**B′**,**B″**, arrow heads). (**C**) Quantitative analysis of average percentage of CSMN expressing 5hmC in hSOD1^G93A^-UeGFP mouse at P120. (**D**) Quantitative analysis of average intensity of 5hmC expression in CSMN of hSOD1^G93A^-UeGFP mouse at P120. (**E**) Representative image of layer V of motor cortex from WT-UeGFP mouse at P150 showing GFP-labelled CSMN and 5hmC expression. Box enlarged (**E′**,**E″**, arrow heads). (**F**) Representative image of layer V of motor cortex from prp-TDP-43^A315T^-UeGFP mouse at P150 showing GFP-labelled CSMN and 5hmC expression. Box enlarged (**F′**,**F″**, arrow heads). (**G**) Quantitative analysis of average percentage of CSMN expressing 5hmC in prp-TDP-43^A315T^-UeGFP mouse at P150. (**H**) Quantitative analysis of average intensity of 5hmC expression in CSMN of prp-TDP-43^A315T^-UeGFP mouse at P150. * *p* < 0.025, ** *p* < 0.001, *** *p* < 0.0005; Scale bar: 20 µm.

Hydroxymethylation levels of CSMN were analyzed in hSOD1^G93A^ and prp-TDP-43^A315T^ mouse models of ALS during end-stage of the disease. Analysis of 5-hydroxymethylcytosine expression was used to assess hydroxymethylation levels in diseased CSMN. CSMN of hSOD1^G93A^ mice showed significantly increased expression of 5hmC when compared to CSMN of healthy controls (WT-UeGFP: 63.5 ± 1.7%; hSOD1^G93A^-UeGFP: 77.6 ± 1.2%, *n* = 3, *p* < 0.002, Figure 2A–C). Interestingly, the intensity of 5hmC expression was also increased in diseased CSMN (WT-UeGFP: 152,016 ± 5466; hSOD1^G93A^-UeGFP: 182,636 ± 1484, *n* = 3, *p* < 0.005, Figure 2D). In striking contrast to CSMN that become diseased due to mSOD1 toxicity, CSMN of prp-TDP-43^A315T^ mouse displayed reduced hydroxymethylation (WT-UeGFP: 68.6 ± 1.3%; prp-TDP-43^A315T^-UeGFP: 19.3 ± 5.1%, *n* = 3, *p* < 0.0005, Figure 2E–E″,G). Measurement of intensity of expression also confirmed reduction in 5hmC expression in CSMN of prp-TDP-43^A315T^ mouse (WT-UeGFP: 237,614 ± 28,228, prpTDP-43^A315T^-UeGFP: 115,764 ± 20,764, *n* = 3, *p* < 0.025, Figure 2H).

Our analyses of 5mC and 5hmC expression were restricted to eGFP expressing CSMN in both hSOD1^G93A^-UeGFP and prp-TDP-43^A315T^–UeGFP mice. 5mC and 5hmC expressions were also observed in other cells adjacent to CSMN. However, the identity of these cells could not be ascertained since we did not perform 5mC and 5hmC co-immunostaining with GFAP (astrocyte), Iba1 (microglia), and NeuN/MAP2 (neuron).

## 4. Discussion

Epigenetic modification of DNA is emerging as a key contributory factor in ALS pathology [30]. In sporadic ALS patients, 5mC expression is increased in the motor cortical neurons, and the elevated 5mC levels are a potential contributor to apoptosis of these neurons [30]. These modifications result in differential DNA methylation that leads to modulation of gene expression in ALS [31]. Whole methylation profiling followed by transcriptomics have identified global gene expression changes in spinal cord and blood cell of ALS patients [23,32]. These gene expression alterations are implicated in various pathways such as immune response and cellular transport [33,34,35]. 

However, majority of these studies investigated the global changes in DNA methylation of either blood or brain and/or spinal cord tissues of ALS patients. Motor neurons degenerate selectively and progressively in ALS. Thus, investigating motor neuron specific DNA methylation, and hydroxymethylation would reveal cell-type-specific vulnerability that might have been masked in global epigenome profiles. Here, we report that CSMN that are diseased due to mSOD1 toxicity and TDP-43 pathology undergo reduction in methylation. In ALS patients, upper as well as lower motor neurons display accumulation of DNA damage response that leads to lower methylation levels in the promoter region of specific genes such as *Ogg1, Apex1, Pnkp,* and *Aptx* in ALS [36]. This phenomenon is also observed in iPS motor neurons derived from familial ALS patients [36]. Recently, reduced methylation levels were observed in mitochondrial DNA regulatory region (D-loop) of ALS-associated genes such as *C9orf72*, *SOD1, FUS*, and *TDP-43* [37]. Differential methylation was reported in *RAD9B* and *C8orf46, CCNF*, *DPP6, RAMP3*, and *CCS* genes in monozygotic twins and triplets discordant for amyotrophic lateral sclerosis [38]. *C9orf72* has been shown to have increased methylation and decreased transcription in ALS/FTD patients with the pathogenic repeat expansion [26,27,39]. Therefore, convincing evidence suggesting a key role of differential DNA methylation in ALS, is beginning to emerge.

We found that CSMN of hSOD1^G93A^ mouse showed higher levels of 5hmC. Hydroxymethylation is critical for postnatal neurodevelopment and aging [40,41]. The presence of 5hmC has generally been associated with increased gene expression [42]. 5hmC-mediated epigenetic regulation is implicated in the development of human cerebellum [43]. In human brains, the promoter region coding for genes involved in ion transport, neuronal development, and cell death has increased 5hmC [42]. Emerging evidence suggests that dysregulation of hydroxymethylation contributes to neurodegenerative diseases including Alzheimer’s, Parkinson’s, Huntington’s disease, and cerebrovascular diseases [44,45]. Selective loss of 5hmC in the cortex and hippocampus of AD patients and 3xTg mouse model promotes AD pathology [46]. Increased levels of hydroxymethylation were observed in hippocampal dentate gyrus, CA3 and CA1–2 regions of aging mice, and caloric restriction helped lower hmC levels [47]. Our results suggest that diseased CSMN acquire hydroxymethylation. Overall, this study reveals that approximately 60% degenerating CSMN lose 5mC expression both in hSOD1^G93A^ and prp-TDP-43^A315T^ mouse. Intriguingly, about 80% degenerating CSMN express 5hmC in hSOD1^G93A^ mouse but about 80% CSMN in prp-TDP-43^A315T^ lose 5hmC expression. CSMN expressing mutated form of hTDP-43 showed reduced levels of 5hmC. Discrepancy in 5hmC expression between hSOD1^G93A^ and prp-TDP-43^A315T^ could be attributed to dysregulation of TDP-43. In a recent study, it was found that neurons that show aberrant localization of TDP-43 into the cytoplasm showed lower levels of 5hmC [24]. Thus, our results suggest that TDP-43 pathology modulates DNA hydroxymethylation specifically in CSMN, and differential expression of 5mC and 5hmC could also be attributed to different underlying causes of CSMN vulnerability [48].

## 5. Conclusions

In summary, this study sheds light on differential DNA methylation and DNA hydroxymethylation specific to CSMN that are vulnerable to degeneration due to different underlying causes in ALS. Further investigation into genes associated with differential methylome, and hydroxymethylome would lead to understanding the basis of selective vulnerability of CSMN in ALS and other upper motor neuron diseases.

## Data Availability

The data presented in this study are contained within the article.
